# Global Burden and Future Projections of Alopecia Areata: A Global Burden of Disease Study 2023 Analysis

**DOI:** 10.3390/jcm15187286

**Published:** 2026-09-19

**Authors:** Aditya K. Gupta, Gregory Pellegrino

**Affiliations:** 1 Division of Dermatology, Department of Medicine, Temerty Faculty of Medicine, University of Toronto, Toronto, ON M5S 1A8, Canada; 2 Division of Dermatology, Women’s College Hospital, Toronto, ON M5S 1B2, Canada; 3 Independent Researcher, London, ON N5Y 4A8, Canada

**Keywords:** alopecia areata, global burden of disease, disease projections, Bayesian age-period-cohort, decomposition analysis, Socio-demographic Index, hair loss, incidence, epidemiology

## Abstract

**Background/Objectives**: Alopecia areata (AA) is a common autoimmune disorder with substantial psychosocial impact, but contemporary assessments of its epidemiology and future burden remain limited. We aimed to comprehensively characterize the epidemiology of AA using the most recent Global Burden of Disease (GBD) 2023 data. **Methods**: GBD 2023 data were used to estimate regional AA incidence. Bayesian age-period-cohort modeling projected incidence through 2040, and decomposition analysis quantified the contributions of population growth, population aging, and epidemiologic change to increases in incident cases from 1990 to 2023. Age-specific incidence patterns were compared across six GBD regions, and associations between the Socio-demographic Index (SDI) and age-standardized incidence rates (ASIRs) were evaluated. **Results**: Marked regional variation in AA burden was observed in 2023, with South Asia accounting for the largest number of incident cases, while Sub-Saharan Africa experienced the greatest historical increase. Projections suggested South Asia, Sub-Saharan Africa, and High-income North America will contribute the largest increases in global AA incidence through 2040. Population growth was the predominant driver of increasing incident cases. Age-specific incidence consistently peaked at 30–34 years and remained highly conserved across regions. Higher SDI was associated with higher regional ASIRs. **Conclusions**: Future increases in AA burden are expected to be driven predominantly by population growth despite relatively stable ASIRs. Although regional disease burden varied substantially, age-specific incidence patterns remained highly conserved, whereas higher socioeconomic development was associated with higher incidence rates. These findings provide an updated global perspective on AA and may inform future epidemiologic research, healthcare planning, and resource allocation.

## 1. Introduction

Alopecia areata (AA) is a chronic, immune-mediated disorder characterized by nonscarring hair loss that affects approximately 2% of the global population [1]. Although AA is not directly associated with increased mortality, its unpredictable clinical course, frequent relapses, and potential progression to extensive hair loss can substantially impair quality of life [2]. Hair loss often affects self-image, social interactions, and emotional well-being, contributing to increased rates of anxiety, depression, and other psychiatric disorders among affected individuals [3,4]. Beyond its psychosocial impact, AA has emerged as an important public health concern because of its considerable healthcare utilization, work productivity losses, and growing global burden [4,5].

Recent epidemiologic studies have improved understanding of the worldwide distribution of AA, although regional patterns of disease burden continue to evolve and remain incompletely characterized [6]. Previous Global Burden of Disease (GBD) studies using older datasets have demonstrated substantial geographic variation in AA incidence and burden [7], but important questions remain regarding the demographic drivers of these regional differences, the stability of age-specific incidence patterns across populations, and the influence of socioeconomic development on disease occurrence. Improved characterization of these factors may provide insight into future healthcare needs and help inform regional resource allocation and planning.

Using data from the GBD 2023 study, we characterized contemporary regional patterns of AA incidence and projected future incident cases through 2040 using Bayesian age-period-cohort (BAPC) modeling. We further quantified the relative contributions of population growth, population aging, and epidemiologic change to historical increases in incident cases using decomposition analysis, characterized age-specific incidence patterns across focal GBD regions, and evaluated the association between regional socioeconomic development and AA incidence using the Socio-demographic Index (SDI). By integrating descriptive epidemiology, age-specific incidence analyses, socioeconomic analyses, epidemiologic projections, and decomposition analysis, this study provides a comprehensive assessment of the evolving global burden of AA and identifies regional patterns that may help guide future epidemiologic research and healthcare planning.

## 2. Materials and Methods

### 2.1. Data Sources

The GBD 2023 study provides standardized estimates of diseases, injuries, and risk factors using data synthesized from multiple sources, including vital registration systems, population-based surveys, disease registries, and published epidemiologic studies [8]. The GBD 2023 database contains annual estimates for 204 countries and territories from 1990 to 2023, which are organized into hierarchical geographic regions. This study examined global estimates and 16 GBD regions representing all 204 countries and territories (Table S1).

Annual incident cases of AA were extracted from the GBD Results Tool at the global and regional levels. Annual disability-adjusted life years (DALYs) of AA, representing disease burden, were also accessed. Age-specific incidence rates (per 100,000 population) of AA from 1990 and 2023 for select GBD regions were also extracted to characterize regional age-specific incidence patterns. Data were stratified by 20 five-year age groups (<5 years to ≥95 years) with both sexes combined. Annual age-standardized incidence rates (ASIRs) for AA were also extracted. The GBD Results Tool is publicly available at https://vizhub.healthdata.org/gbd-results/ (accessed on 14 July 2026) [8].

Population data were obtained from the United Nations Department of Economic and Social Affairs, Population Division, World Population Prospects 2024 [9]. Annual population estimates for 1950–2023 and median population projections for 2024–2100 were extracted for both sexes combined in corresponding five-year age groups. Population estimates for individual countries were subsequently aggregated to match the composition of the 16 GBD regions used in this study. The World Population Prospects database is publicly available at https://population.un.org/wpp/downloads?folder=Standard%20Projections&group=Population (accessed on 26 May 2026) [9].

Annual SDI values for all GBD 2023 locations from 1950 to 2023 were also accessed for analysis. All SDI values are available at https://ghdx.healthdata.org/record/gbd-2023-socio-demographic-index-sdi (accessed on 31 July 2026) [10].

### 2.2. Bayesian Age-Period-Cohort (BAPC) Prediction Modeling

Future incident cases from 2024 to 2040 were projected using a BAPC model. Age-specific incident case counts and population estimates were arranged into annual age-period matrices comprising 20 age groups (<5 years to ≥95 years, in 5-year intervals) and 51 calendar years (1990–2040). Observed incident case counts and population estimates were available for 1990–2023, whereas population projections for 2024–2040 were obtained from United Nations population forecasts [9]. Because the BAPC framework models count data, incident case estimates were rounded to the nearest integer before model fitting.

The BAPC model decomposes disease occurrence into age, period, and cohort effects while accounting for uncertainty within a Bayesian framework. Projections were generated using integrated nested Laplace approximation (INLA), which provides computationally efficient Bayesian inference and estimation of predictive uncertainty [11,12]. Annual projections were generated for 2024–2040, and projected age-specific incident cases were subsequently aggregated to estimate the total number of incident cases for each year. For projected incident cases, 95% uncertainty intervals (UIs) were calculated as the 2.5th and 97.5th percentiles of 5000 samples drawn from the joint posterior distribution of each fitted model.

Six regions were selected post hoc for detailed analysis based on regional patterns in disease incidence and projected changes, with the aim of capturing regions exhibiting particularly high incidence or notable projected increases. Predictive performance of the BAPC models was retrospectively validated for the global model and the six selected regions. The final five years of observed data (2019–2023) were withheld, models were fitted using data from 1990 to 2018, and predictions for the withheld period were compared with observed GBD estimates (Table S2).

To characterize changes in regional contributions to the global burden, the percentage contribution of each GBD region to the increase in global incident cases was calculated for both the historical (1990–2023) and projected (2023–2040) periods. Regional contributions were calculated by dividing the absolute increase in incident cases for each region by the corresponding global increase in incident cases and multiplying by 100. Regions were classified as exhibiting increasing, decreasing, or stable contributions according to whether the projected contribution differed from the historical contribution by more than ±1 percentage point. Regions with projected contributions within ±1 percentage point of their historical contribution were classified as having stable contributions.

Age-standardized incidence rates (ASIRs) were calculated to facilitate comparisons across populations and over time by accounting for differences in age structure and population size. For the observed period (1990–2023), age-specific incidence rates were calculated by dividing age-specific incident cases by corresponding age-specific population estimates. For the projected period (2024–2040), age-specific projected incident cases from the BAPC model were divided by the corresponding projected age-specific population estimates to obtain projected age-specific incidence rates. Age-specific incidence rates for both observed and projected periods were then multiplied by the GBD standard population age weights, summed across all age groups, and multiplied by 100,000 to generate annual ASIRs expressed per 100,000 population, both globally and per region [13]. For projected ASIRs, 95% UIs were obtained from the corresponding BAPC age-standardized rate posterior estimates.

### 2.3. Decomposition Analysis

Decomposition analyses were performed to quantify the contributions of population growth, population aging, and epidemiologic change to the increase in alopecia areata incident cases between 1990 and 2023. Changes in incident cases were partitioned into components attributable to changes in total population size, population age structure, and age-specific incidence rates. In this framework, population growth represents changes resulting solely from increases or decreases in population size, whereas population aging reflects changes attributable to shifts in the population age structure. Epidemiologic change represents changes in age-specific incidence rates after accounting for changes in population size and age structure, thereby reflecting changes driven by factors such as (but not limited to) disease occurrence, healthcare access, diagnostic practices, and underlying risk factor exposure.

Total incident cases were expressed as the sum, across age groups, of the product of the total population size, the age-specific population proportion (representing age structure), and the corresponding age-specific incidence rate. A stepwise counterfactual decomposition approach was used to partition the observed change in incident cases into three components. First, the contribution of population growth was estimated by replacing the 1990 total population with the 2023 total population while holding the 1990 age structure and age-specific incidence rates constant. Next, the contribution of population aging was estimated by replacing the 1990 age structure with the 2023 age structure, while retaining the 2023 total population and the 1990 age-specific incidence rates. Finally, the contribution of epidemiologic change was estimated by replacing the 1990 age-specific incidence rates with the observed 2023 age-specific incidence rates, while retaining the 2023 total population and 2023 age structure. The differences between successive counterfactual scenarios were interpreted as the contributions of population growth, population aging, and epidemiologic change, respectively.

### 2.4. Age-Specific Incidence Analysis

Age-specific incidence rates for AA were extracted for the six GBD regions that were selected for further analysis following the initial regional analyses (High-income North America, Western Europe, South Asia, Southeast Asia, East Asia, and Oceania, North Africa and the Middle East, and Sub-Saharan Africa) for 1990 and 2023. Incidence rates were stratified into 20 five-year age groups (<5 years to ≥95 years) to characterize regional age-specific incidence patterns and identify age groups with the highest incidence. Pairwise Pearson correlation coefficients were calculated between the age-specific incidence profiles of each of the six GBD regions to quantify the similarity of age-specific incidence profiles between regions in each study year.

### 2.5. Socio-Demographic Index Analysis

The Socio-demographic Index (SDI) is a composite measure of socioeconomic development used in the GBD study that incorporates lag-distributed income per capita, average educational attainment among individuals aged 15 years and older, and the total fertility rate among women younger than 25 years. SDI ranges from 0 to 1, with higher values indicating greater socioeconomic development [8]. Annual SDI estimates for the 16 study regions were merged with corresponding regional ASIRs for 1990–2023. To distinguish persistent between-region differences in socioeconomic development from temporal changes within regions, a hybrid linear mixed-effects model was fitted with region included as a random intercept. Regional mean SDI (between-region effect) and deviations from each region’s mean SDI (within-region effect) were included simultaneously as fixed effects, allowing simultaneous estimation of between-region and within-region associations between SDI and ASIR. Residual temporal autocorrelation within regions was accounted for using a first-order autoregressive [AR(1)] correlation structure. SDI predictors were scaled such that model coefficients represent associations per 0.1-unit increase in SDI. Additionally, mean SDI and mean ASIR were calculated for each region over the study period, and their association was assessed using Spearman rank correlation.

### 2.6. Statistical Analyses

All analyses and graphical representations were conducted with the statistical computing software R (version 4.6.0) [14]. Bayesian age-period-cohort projections were performed using the BAPC (v0.0.37) and INLA (v25.10.19) packages [11,12]. Hybrid linear mixed-effects models were fitted using the nlme package (v3.1.169) [15,16]. Data management and graphical representations were performed using packages from the tidyverse, including packages dplyr v(1.2.1), tidyr (v1.3.2), stringr (1.6.0), and ggplot2 (v4.0.3) [17]. Geographic figures were generated using packages sf (v1.1.1), rnaturalearth (v1.2.0), and lwgeom (v0.2.16) [18,19,20].

## 3. Results

### 3.1. Global Distribution and Temporal Trends in Alopecia Areata Incidence

**Figure 1:**
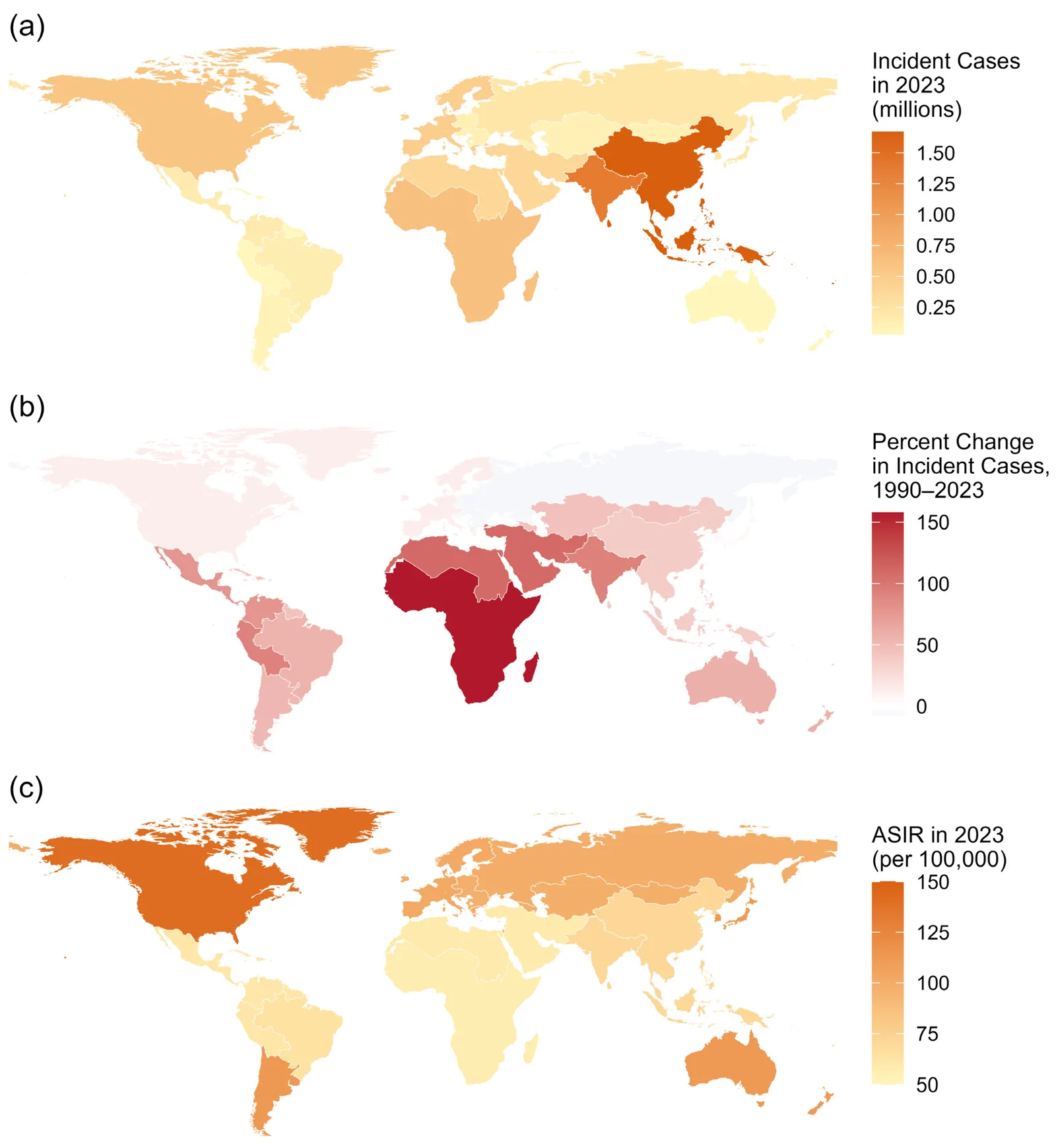
Regional distribution and change in alopecia areata incidence. (**a**) Estimated incident cases of alopecia areata (AA) in 2023 by Global Burden of Disease (GBD) region, expressed in millions of cases. (**b**) Percent change in incident cases between 1990 and 2023 by GBD region. (**c**) Age-standardized incidence rates (ASIRs) per 100,000 population in 2023 by GBD region.

The global distribution of AA incident cases varied substantially across GBD regions in 2023 (Figure 1a, Table S3). The greatest burden was observed in the combined Southeast Asia, East Asia, and Oceania region (1.67 million incident cases), followed by South Asia (1.38 million), and Sub-Saharan Africa (0.62 million). In contrast, the Caribbean had the lowest number of incident cases (0.03 million).

Between 1990 and 2023, AA incident cases increased in most regions, although the magnitude of change varied considerably (Figure 1b). The largest absolute increases were observed in South Asia (+664,632 cases), the Southeast Asia, East Asia, and Oceania region (+458,932 cases), and Sub-Saharan Africa (+383,023 cases). Relative increases were greatest in Sub-Saharan Africa (+157.6%), North Africa and the Middle East (+109.8%), and South Asia (+92.8).

In contrast, incident cases declined slightly in Central Europe (−7.1%) and Eastern Europe (−7.5%). While High-income North America and Western Europe both had relatively high total incident cases in 2023 (0.57 and 0.48 million, respectively), both also demonstrated comparatively modest increases from 1990 to 2023 (12.1% and 12.8%, respectively).

Age-standardized incidence rates (ASIRs) also demonstrated substantial regional variation in 2023 (Figure 1c). High-income North America had the highest ASIR (141.5 cases per 100,000 population), followed by Southern Latin America (112.4), Australasia (111.7), and High-income Asia Pacific (111.4). In contrast, the lowest ASIRs were observed in Sub-Saharan Africa (57.1), North Africa and the Middle East (58.6), the Caribbean (61.5), and Andean Latin America (61.5). Overall, ASIRs tended to be highest in high-income regions and lowest across much of Africa and Latin America.

Overall, these findings indicate that the contemporary global burden of AA is concentrated primarily in Asia, whereas the most rapid historical growth has occurred in Africa.

### 3.2. Historical and Projected Regional Contributions to Global Incidence

**Figure 2:**
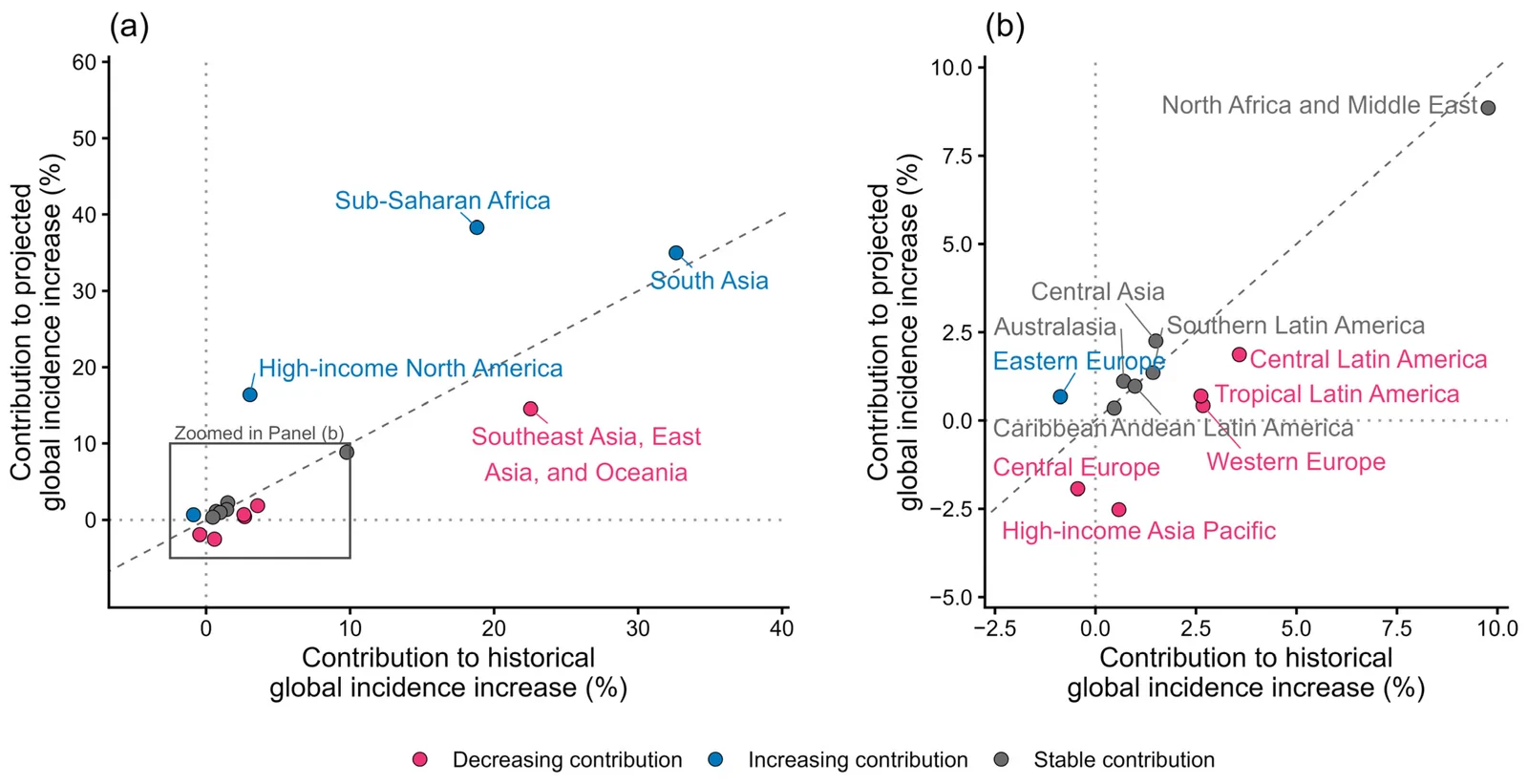
Historical and projected regional contributions to the global increase in alopecia areata incidence. (**a**) Scatterplot comparing each GBD region’s contribution to the historical global increase in incident cases (1990–2023; *x*-axis) with its projected contribution to the future global increase in incident cases (2023–2040; *y*-axis). Regions above the diagonal line are projected to contribute proportionally more to future increases than historically, whereas regions below the line are projected to contribute proportionally less. Colors indicate regions with increasing (pink), decreasing (blue), or stable (gray) relative contributions. (**b**) Enlarged view of the clustered regions highlighted in panel (**a**).

BAPC projections were performed to estimate global and regional AA incident cases from 2024 to 2040. Regional contributions to the global increase in incident cases were projected to shift between the historical period (1990–2023) and the projection period (2023–2040) (Figure 2, Table S3). Globally, incident cases rose from approximately 4.24 million in 1990 to 6.27 million in 2023 and were projected to reach 7.19 million (95% UI, 5.41–9.37 million) by 2040. South Asia remained a major contributor to the global increase, accounting for 32.6% of the historical increase and 35.0% of the projected increase. Incident cases in South Asia were projected to rise from 1.38 million in 2023 to 1.70 million (95% UI, 1.32–2.16 million) in 2040.

The largest change in relative contribution was observed in Sub-Saharan Africa, whose share was projected to rise from 18.8% historically to 38.3% during the projection period, corresponding to an increase from approximately 626,000 incident cases in 2023 to 978,000 (95% UI, 754,000–1,239,000) in 2040. High-income North America also showed a greater projected contribution, increasing from 3.1% to 16.4%, with incident cases expected to rise from approximately 574,000 to 724,000 (95% UI, 156,000–2,083,000). Eastern Europe was also projected to have a very minor increase in contribution to global incident cases. North Africa and the Middle East remained a substantial but relatively stable contributor, declining slightly from 9.8% to 8.9%.

In contrast, the Southeast Asia, East Asia, and Oceania region showed the largest reduction in relative contribution, with a predicted decrease from 22.5% to 14.5%. Nevertheless, the region remained one of the largest contributors to global AA incidence, as incident cases were projected to increase from 1.67 million in 2023 to 1.80 million (95% UI, 1.36–2.36 million) in 2040.

Decreased projected contributions were also observed for High-income Asia Pacific, Western Europe, Central Europe, Central Latin America, and Tropical Latin America. Most other regions remained clustered near the line of equality, indicating comparatively modest changes in their shares of global incidence growth. Overall, the projected global burden of AA is expected to become increasingly concentrated in South Asia and Sub-Saharan Africa, underscoring the importance of understanding the demographic drivers underlying these regional differences.

**Figure 3:**
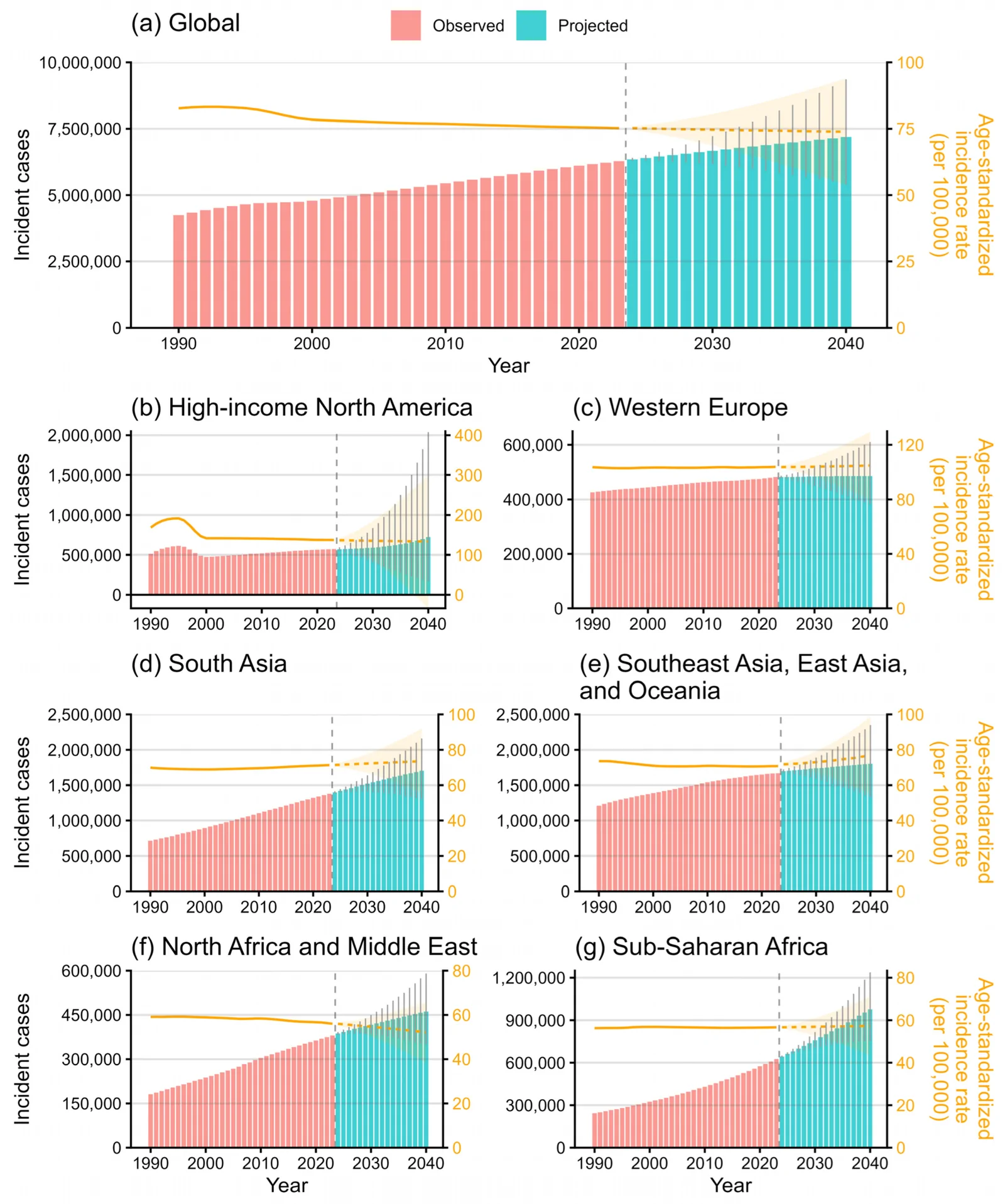
Trends and projections of alopecia areata incidence, 1990–2040. (**a**) Global estimates of incident cases and age-standardized incidence rates, and (**b**–**g**) corresponding estimates for selected GBD regions: (**b**) High-income North America, (**c**) Western Europe, (**d**) South Asia, (**e**) Southeast Asia, East Asia, and Oceania, (**f**) North Africa and Middle East, and (**g**) Sub-Saharan Africa. Bars represent annual incident cases (left y-axis), and the orange line represents the age-standardized incidence rate per 100,000 population (right y-axis). The orange shaded band and gray error bars represent 95% uncertainty intervals (UIs) for projected ASIRs and incident cases, respectively. Pink bars and solid orange lines indicate observed estimates from 1990 to 2023, whereas teal bars and dashed orange lines indicate BAPC projections for 2024–2040. The vertical dashed line denotes the transition from observed to projected estimates.

Based on these findings, six focal GBD regions were selected for further analysis because they exhibited either a high burden of incident cases or made substantial contributions to historical or projected changes in global incidence. Figure 3 illustrates historical (1990–2023) and projected (2024–2040) trends in incident cases and age-standardized incidence rates (ASIRs) globally and across these regions. Globally, incident cases were projected to continue increasing through 2040, whereas the ASIR was projected to remain relatively stable. Similar patterns were observed across all selected regions, although the magnitude of projected increases varied considerably. The greatest future increases in incident cases were projected for South Asia, Sub-Saharan Africa, High-income North America, and Southeast Asia, East Asia, and Oceania, whereas Western Europe and North Africa and Middle East exhibited more modest increases. Absolute values of ASIRs were highest in High-income North America and Western Europe throughout the study period. ASIRs were projected to decrease in High-income North America and North Africa and the Middle East, increase in South Asia and Southeast Asia, East Asia, and Oceania, and remain relatively stable in Western Europe and Sub-Saharan Africa. Across all regions, projected changes in incident cases were substantially greater than changes in ASIRs. BAPC projections were additionally conducted on AA disability-adjusted life years (DALYs), to examine disease burden rather than incidence; regional patterns remained consistent between AA incidence and AA burden (Figure S1).

### 3.3. Demographic Drivers of Incidence and Age-Specific Incidence Patterns

**Figure 4:**
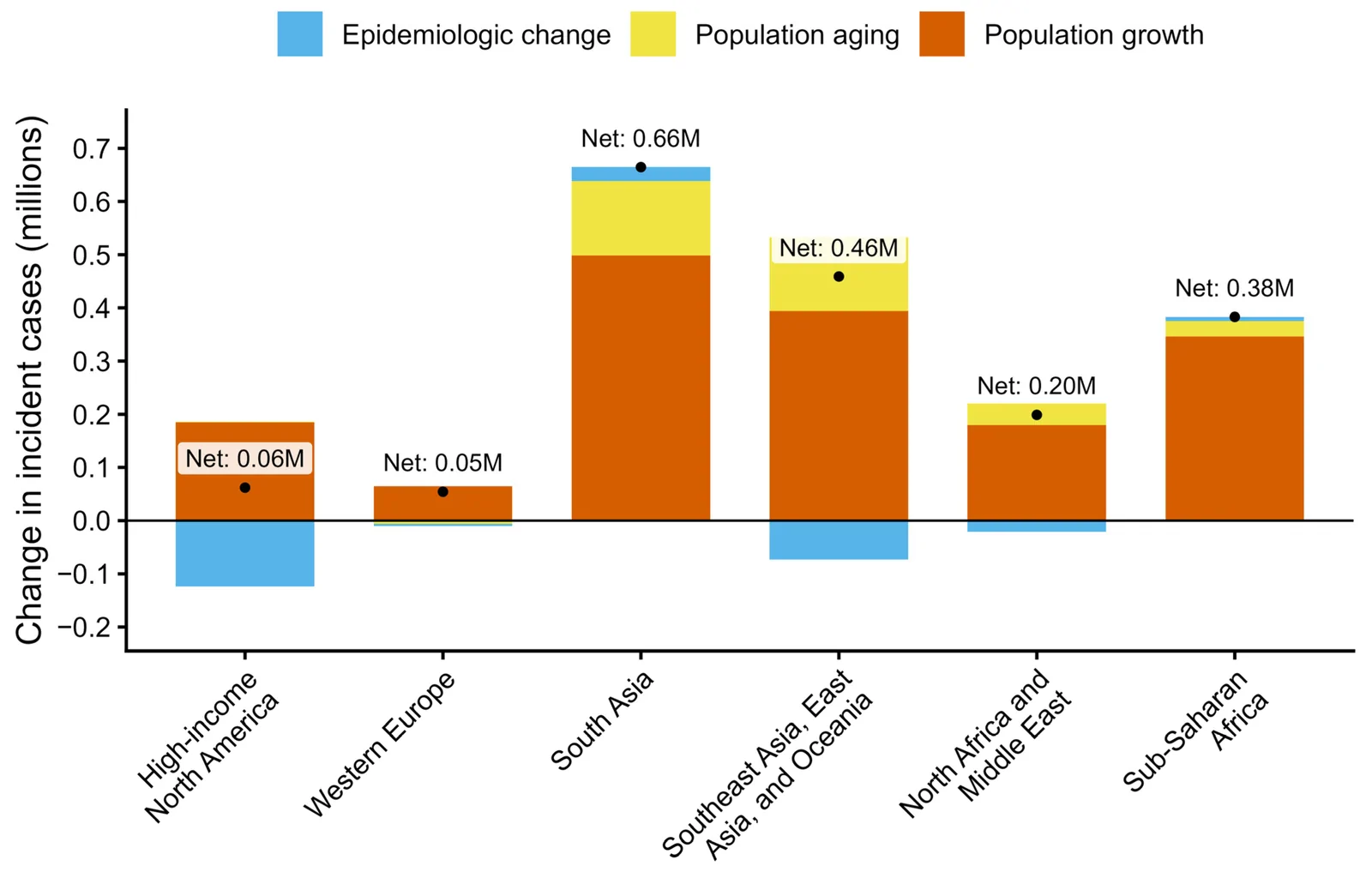
Decomposition of changes in alopecia areata incident cases by demographic and epidemiologic drivers, 1990–2023. Stacked bars show the contributions of population growth, population aging, and epidemiologic change to the overall change in incident cases for the six selected GBD regions. Positive values indicate factors contributing to an increase in incident cases, whereas negative values indicate factors offsetting this increase. Black points and labels denote the net change in incident cases for each region over the study period.

Decomposition analysis of the six selected regions demonstrated that population growth was the principal driver of increasing AA incident cases from 1990 to 2023 (Figure 4, Table S4). The largest net increase in incident cases was observed in South Asia (+0.66 million), followed by Southeast Asia, East Asia, and Oceania (+0.46 million), Sub-Saharan Africa (+0.38 million), North Africa and the Middle East (+0.20 million), High-income North America (+0.06 million), and Western Europe (+0.05 million).

Population growth accounted for the majority of the increase in incident cases in every region, contributing approximately 499,000 additional cases in South Asia, 394,000 in Southeast Asia, East Asia, and Oceania, 346,000 in Sub-Saharan Africa, and 179,000 in North Africa and the Middle East. Population aging also contributed positively to increasing incidence in most regions, particularly in Southeast Asia, East Asia, and Oceania (+138,000 cases) and South Asia (+140,000 cases), although its overall contribution was substantially smaller than that of population growth.

The contribution of epidemiologic change varied across regions. Epidemiologic change was associated with reductions in incident cases in High-income North America (−124,000 cases), Southeast Asia, East Asia, and Oceania (−73,000 cases), North Africa and Middle East (−21,000 cases), and Western Europe (−3800 cases), partially offsetting demographic increases in these regions. In contrast, epidemiologic change contributed modest increases in incident cases in South Asia (+26,000 cases) and Sub-Saharan Africa (+7900 cases). Overall, these findings indicate that observed increases in AA incidence between 1990 and 2023 were driven predominantly by demographic change, particularly population growth, rather than changes in the underlying epidemiology of the disease.

**Figure 5:**
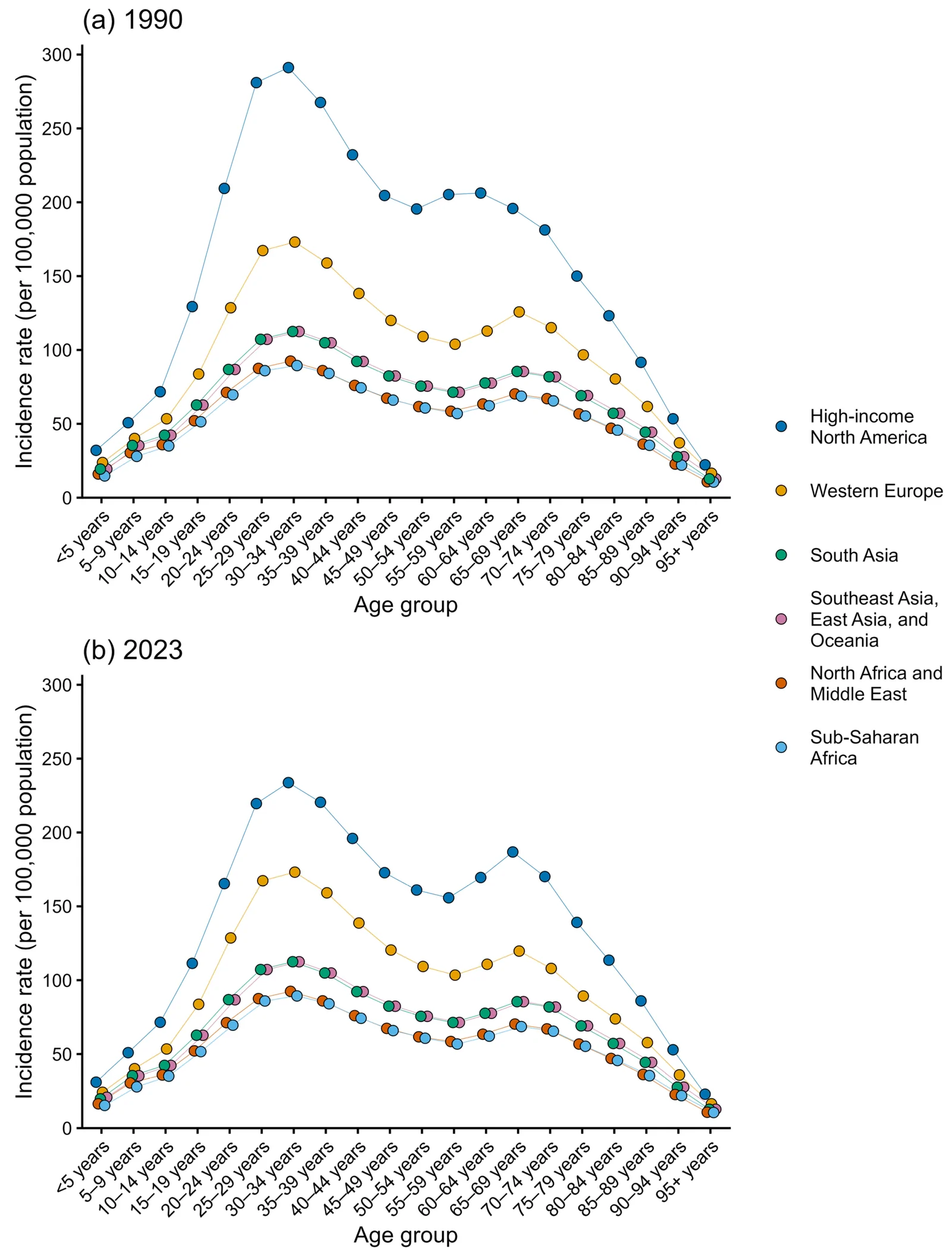
Age-specific incidence rates of AA in focal GBD regions. Incidence rates are shown per 100,000 population for both sexes in (**a**) 1990 and (**b**) 2023. Across all six regions, incidence increased from childhood, peaked at 30–34 years of age, and subsequently declined with increasing age, with a modest secondary increase observed around 65–69 years.

Age-specific incidence patterns were broadly consistent across the six selected GBD regions in both 1990 and 2023 (Figure 5). In all regions, incidence increased progressively from childhood, peaked in adults aged 30–34 years, and declined thereafter with increasing age. A smaller secondary increase in incidence was observed in the 65–69-year age group. High-income North America exhibited the highest incidence rates across nearly all age groups in both years, followed by Western Europe, whereas South Asia, Southeast Asia, East Asia, and Oceania, North Africa and the Middle East, and Sub-Saharan Africa showed similar age-specific patterns with lower overall incidence. Despite absolute differences in incidence rates, the age-incidence distribution was highly conserved across regions, with pairwise Pearson correlation coefficients ranging from 0.983 to 1.000 in 1990 and 0.988 to 1.000 in 2023 (Table S5).

### 3.4. Association Between Socio-Demographic Index and Age-Standardized Incidence Rates

**Figure 6:**
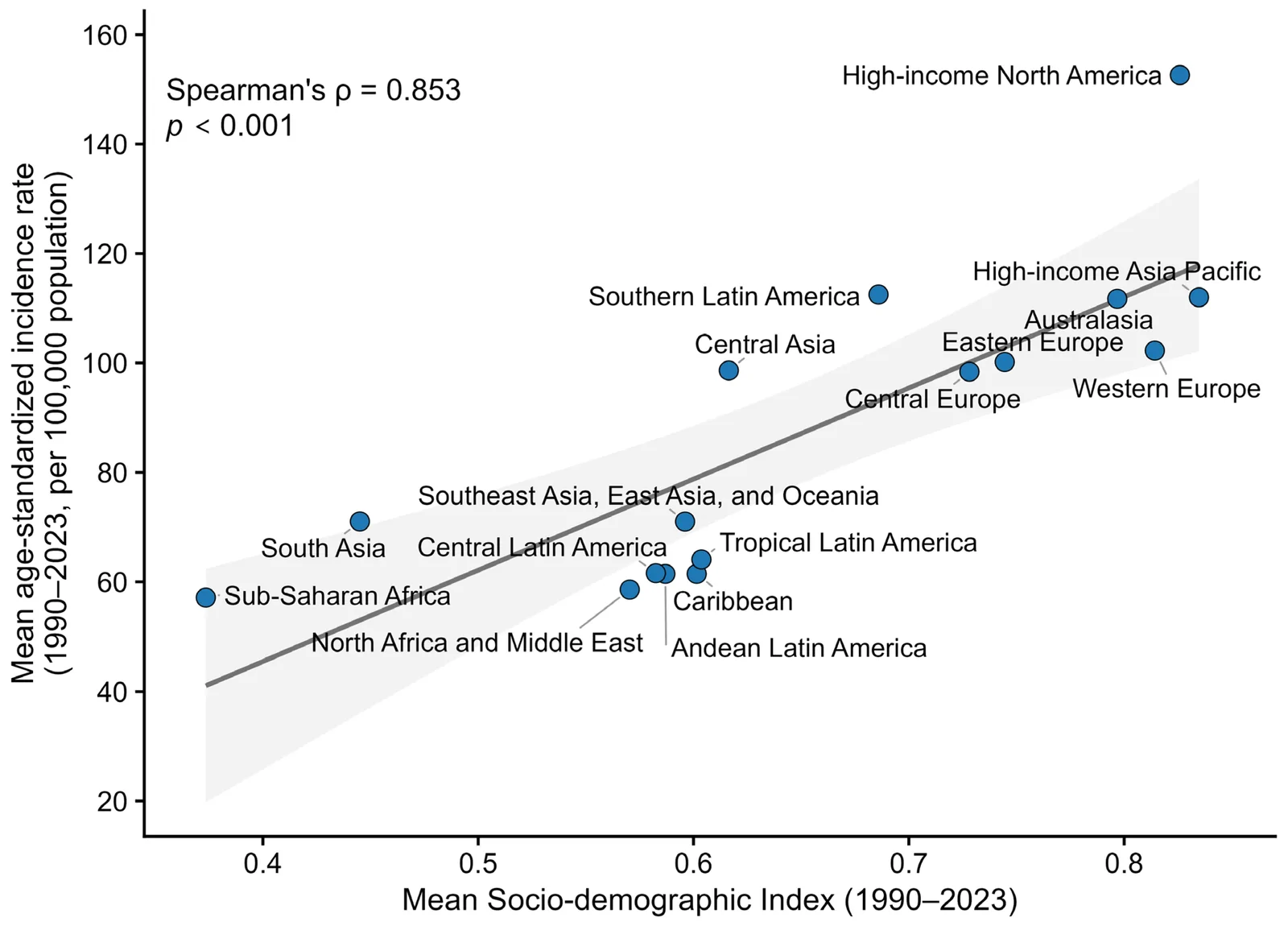
Association between mean Socio-demographic Index (SDI) and mean age-standardized incidence rate (ASIR) of AA across 16 GBD regions, 1990–2023. Each point represents one GBD region plotted according to its mean SDI and mean ASIR over the study period. The solid line represents the fitted linear regression, and the shaded area indicates the 95% confidence interval. Spearman’s rank correlation coefficient (ρ) and associated *p*-value are shown.

Mean regional AA ASIR was strongly positively correlated with mean SDI over the study period (Spearman’s ρ = 0.853, *p* < 0.001) (Figure 6). Regions with the highest average SDI, including High-income North America, High-income Asia Pacific, and Western Europe, also exhibited the highest mean ASIRs, whereas Sub-Saharan Africa had both the lowest mean SDI and the lowest mean ASIR.

Hybrid mixed-effects regression separating between-region and within-region SDI effects and accounting for temporal autocorrelation demonstrated that higher mean regional SDI was independently associated with higher ASIR, whereas changes in SDI within regions over time were not significantly associated with ASIR (Table 1).

## 4. Discussion

In this global analysis of GBD 2023 data, we characterized contemporary regional patterns of AA incidence, projected regional contributions to future global AA incidence through 2040 using BAPC modeling, quantified the demographic drivers underlying historical changes in incident cases, examined age-specific incidence patterns across focal GBD regions, and quantified the association between socioeconomic development and AA incidence. To our knowledge, this is the first study using the most recent GBD 2023 data to integrate these analyses into a single comprehensive assessment of AA. Our findings demonstrate substantial regional heterogeneity in both current and projected AA burden. High-income North America, South Asia, and Sub-Saharan Africa were projected to account for an increasing share of future global incidence, while decomposition analyses demonstrated that population growth was the primary driver of increasing incident cases across selected GBD regions. In addition, age-specific incidence patterns remained remarkably consistent across regions, and higher socioeconomic development was associated with higher AA incidence rates.

High-income North America was a notable outlier identified from the projections of future contributions to global incident cases, with an increasing contribution to global incident cases despite its high-income status. In contrast, other high-income regions with slower population growth or population stabilization, such as Western Europe, are projected to contribute a progressively smaller proportion of global incident cases. The projected shift in the global burden of AA toward South Asia and Sub-Saharan Africa appears to be driven predominantly by demographic factors, as both regions are expected to experience sustained population growth over the coming decades, increasing the absolute number of individuals at risk [9].

The regional BAPC projections provide additional insight into the evolution of AA incidence over time. Across all selected regions, incident cases were projected to continue increasing through 2040, whereas annual age-standardized incidence rates (ASIRs) remained comparatively stable. The relative stability of ASIRs across diverse geographic regions suggests that this pattern is broadly consistent despite marked differences in the absolute burden of disease. The divergence between incident cases and ASIRs suggests that future increases in AA burden are likely to reflect demographic changes, including population growth and aging, rather than substantial increases in age-specific disease risk. However, our finding that higher regional SDI was associated with higher AA incidence suggests that socioeconomic differences may also contribute to geographic variation in reported incidence. Whether these differences reflect true variation in disease occurrence, improved case ascertainment, or a combination of both remains uncertain.

Of the six regions examined in depth, High-income North America and Western Europe consistently exhibited the highest ASIRs throughout the study period. Similarly, regional ASIRs in 2023 were highest in high-income regions, and the positive association between regional SDI and ASIRs further supports these observations. Regions with higher average SDI consistently exhibited higher incidence rates, and hybrid mixed-effects modeling accounting for temporal autocorrelation demonstrated a strong positive between-region association between SDI and ASIR. In contrast, changes in SDI over time within individual regions were not significantly associated with ASIR. These findings suggest that the higher ASIRs observed in high-SDI regions are associated with persistent differences between regions rather than increases in socioeconomic development occurring within regions over time.

Although these findings may reflect true regional differences in disease occurrence, they may also partly reflect greater access to healthcare, higher dermatologist density, increased disease awareness, and more complete case ascertainment in higher-SDI settings. The absence of a significant within-region association further suggests that the higher incidence observed in high-SDI regions should not be interpreted as evidence that increasing socioeconomic development itself leads to increasing AA incidence. Our finding of a positive association between regional SDI and AA incidence is consistent with previous analyses using earlier GBD releases that observed higher AA burden in regions with greater socioeconomic development [7]. Conversely, underdiagnosis and reduced access to dermatologic care in lower-resource settings may contribute to lower reported incidence rates. Consequently, regional differences in ASIR should be interpreted in the context of variation in healthcare access and diagnostic practices. Collectively, these findings underscore the importance of anticipating demographic change when planning future dermatologic healthcare services and resource allocation.

The decomposition analysis further supports the interpretation that demographic change is the principal driver of the increasing global burden of AA. Across all study regions, population growth accounted for the largest proportion of the increase in incident cases, whereas population aging contributed more modestly. In contrast, epidemiologic change generally accounted for a comparatively small proportion of the observed increase and, in several regions, contributed negatively to overall incidence, suggesting that changes in disease epidemiology contributed relatively little to the increasing burden of AA after accounting for demographic change. These findings are consistent with recent GBD analyses, which similarly identified population growth as the predominant contributor to the increasing burden of AA, with population aging representing a secondary driver [21]. The present study extends this previous work through updated GBD 2023 estimates and projections to 2040, regional decomposition of changes in incident cases, cross-regional comparison of age-specific incidence patterns, and modeling of distinct between- and within-region associations between SDI and AA incidence.

The contribution of population aging, representing changes in population age structure independent of overall population growth, also differed substantially among regions, reflecting differences in demographic transition. Population aging accounted for a relatively large proportion of the increase in incident cases in South Asia and Southeast Asia, East Asia, and Oceania, where declining fertility and increasing life expectancy have shifted population age distributions toward adulthood and older ages [22,23]. Because AA incidence remains relatively high across much of adulthood, these demographic shifts increased the expected number of incident cases even when age-specific incidence rates were held constant. In contrast, population aging contributed less in Sub-Saharan Africa, where rapid population growth remains the dominant demographic process, and populations remain comparatively young [24]. Similarly, the negative contribution of population aging in Western Europe likely reflects a population that has already undergone demographic transition, with continued but comparatively less dramatic age-structure change. These findings emphasize that the increasing burden of AA is shaped not only by population size but also by evolving regional age structures.

Age-specific incidence analyses demonstrated a highly conserved age distribution of AA across geographically diverse regions. In both 1990 and 2023, incidence consistently peaked among adults aged 30–34 years, and the overall shape of the age-incidence curve remained remarkably similar despite substantial regional differences in absolute incidence. This consistency suggests that the timing of AA onset is relatively stable across populations and that regional differences in disease burden are driven primarily by differences in incidence magnitude rather than shifts in the age groups most commonly affected. The observed peak incidence among adults aged 30–34 years is broadly consistent with previous population-based studies, which have reported the highest AA incidence in adults aged 25–29 years in the United Kingdom and the highest prevalence among adults aged 30–39 years in the United States [25,26]. These findings have practical implications for healthcare planning, as they indicate that diagnostic services, patient education, and specialist dermatologic care should continue to focus predominantly on young adults regardless of geographic setting.

An important consideration when interpreting our projections is the rapidly evolving therapeutic landscape for AA. The BAPC model is based on historical trends and therefore assumes that future disease patterns will largely reflect those observed during the study period. However, the recent introduction of targeted Janus kinase (JAK) inhibitors represents a major advance in AA management. Since the approval of baricitinib in 2022, followed by ritlecitinib in 2023 and deuruxolitinib in 2024, randomized clinical trials have demonstrated substantial and sustained hair regrowth in many patients with moderate-to-severe AA, fundamentally altering the treatment paradigm [27,28]. Broader access to effective treatments may reduce disease severity, disability, and psychosocial burden, potentially mitigating the future health impact of AA beyond that projected from historical epidemiologic trends.

Nevertheless, equitable access to these therapies remains an important challenge. JAK inhibitors are costly, and differences in regulatory approval, healthcare infrastructure, reimbursement policies, and medication affordability may limit their availability in many low- and middle-income countries [29,30]. Consequently, disparities in access to these therapies may limit their population-level impact in some low- and middle-income settings, including regions projected to experience the greatest future burden of AA, thereby risking further widening of global inequities in AA care. Continued surveillance using future GBD iterations will therefore be important to determine whether widespread adoption of these therapies ultimately modifies the global burden of AA.

Several limitations should be considered when interpreting these findings. First, all analyses were based on GBD 2023 estimates, which rely on statistical modeling to harmonize data from multiple sources and are therefore subject to the quality and availability of underlying data. Consequently, estimates may be less precise in regions with limited surveillance or incomplete reporting. Second, the SDI analysis was ecological in nature and based on region-level rather than individual-level data. Consequently, the observed association between socioeconomic development and AA incidence should not be interpreted as evidence of an individual-level causal relationship. Third, historical incidence data for High-income North America demonstrated an anomalous increase during the early 1990s, driven primarily by estimates from the United States. Although this feature may have influenced the projected trends, it was retained because it originated from the published GBD 2023 estimates, and there was no objective justification for selectively excluding these observations. Notably, projections for High-income North America were accompanied by wide uncertainty intervals, indicating substantial uncertainty regarding the magnitude and trajectory of future incidence. Accordingly, these projections should be interpreted with particular caution, precluding firm conclusions regarding the future burden of AA in High-income North America. Finally, sex-stratified analyses were beyond the scope of the present study, which focused on characterizing geographic and temporal patterns in AA incidence and examining factors associated with or contributing to these patterns. Sex-specific differences in AA epidemiology have been characterized in previous GBD-based studies [21], including recent analyses specifically examining variation in AA risk by sex [31].

Despite these limitations, the present study has several notable strengths. First, it integrates multiple complementary analytical approaches, including descriptive epidemiology, BAPC projections, decomposition analysis, and SDI association analyses, providing a comprehensive assessment of contemporary and future regional patterns of AA. Second, by utilizing the most recent GBD 2023 estimates, this study provides an updated evaluation of the global burden of AA and extends previous work through projections to 2040. Third, the use of standardized GBD methodology enabled consistent comparisons across countries and regions despite substantial differences in demographic structure. Finally, the inclusion of decomposition analyses together with age-specific and socioeconomic analyses provides additional context for interpreting projected changes in disease burden and highlights geographic heterogeneity that may inform future research and healthcare planning.

## 5. Conclusions

Using the most recent GBD 2023 estimates, this study provides a comprehensive assessment of the contemporary and future global burden of AA. Although ASIRs are projected to remain relatively stable through 2040, the absolute number of incident cases is expected to increase substantially, driven primarily by population growth. Despite marked regional differences in disease burden, age-specific incidence patterns remained highly conserved across focal GBD regions, whereas higher socioeconomic development was associated with higher incidence rates. These findings provide an updated global perspective on AA and may inform future epidemiologic research, healthcare planning, resource allocation, and strategies to address the growing demand for dermatologic care.

## Figures and Tables

**Table 1:** Hybrid mixed-effects regression examining the association between Socio-demographic Index (SDI) and age-standardized incidence rates of AA across 16 GBD regions, 1990–2023.

Predictor	Beta per 0.1-Unit SDI	SE ^1^	95% CI ^1^	*p*-Value
Between-region SDI ^1^	16.79	3.33	9.65 to 23.93	<0.001
Within-region SDI deviation	−0.49	0.97	−2.39 to 1.41	0.610

^1^ CI, confidence interval; SDI, socio-demographic index; SE, standard error.

## Data Availability

The data analyzed in this study are publicly available through the Global Burden of Disease Results Tool, provided by the Institute for Health Metrics and Evaluation, and through the United Nations Department of Economic and Social Affairs, Population Division, World Population Prospects 2024.

## Supplementary Materials

The following supporting information can be downloaded at https://www.mdpi.com/article/10.3390/jcm15187286/s1. Table S1: Global Burden of Disease (GBD) regional classification of countries; Table S2: Retrospective validation of Bayesian age-period-cohort model predictive performance; Table S3: Incidence patterns by geographical Global Burden of Disease (GBD) Region; Table S4: Decomposition results of change in incident cases, 1990–2023; Table S5: Pairwise Pearson correlation coefficients describing similarity of age-specific incidence profiles between six Global Burden of Disease (GBD) regions in 1990 and in 2023; Figure S1: Trends and projections of alopecia areata burden, 1990–2040.

## Abbreviations

The following abbreviations are used in this manuscript:

AA	Alopecia areata
AR(1)	First-order autoregressive
ASIR	Age-standardized incidence rate
BAPC	Bayesian age-period-cohort
CI	Confidence interval
DALY	Disability-adjusted life year
GBD	Global Burden of Disease
INLA	Integrated nested Laplace approximation
JAK	Janus kinase
SDI	Socio-demographic index
SE	Standard error
UI	Uncertainty interval
