# Supplementary material for: Global Burden and Future Projections of Alopecia Areata: A Global Burden of Disease Study 2023 Analysis
Source: J Clin Med. 2026 Sep 19;15(18):7286. doi: 10.3390/jcm15187286 (PMC13608508; doi:10.3390/jcm15187286)
Supplement: Supplementary file 1 [file jcm-15-07286-s001.zip › Figure S1.pdf]

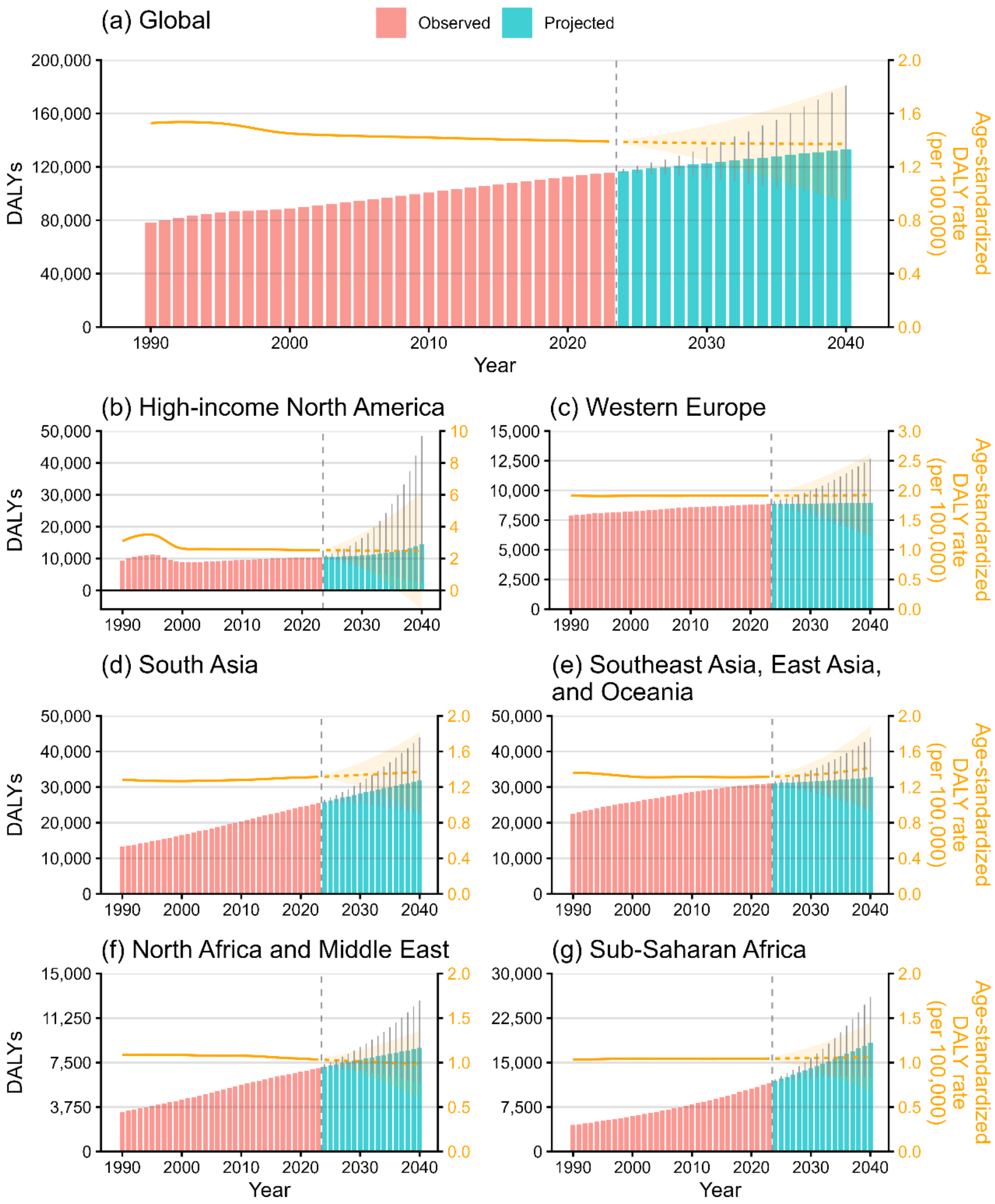

Figure S1. Trends and projections of alopecia areata burden, 1990–2040. (**a**) Global estimates of disability-adjusted life years (DALYs) and age-standardized DALY rates, and (**b-g**) corresponding estimates for selected GBD regions: (**b**) High-income North America, (**c**) Western Europe, (**d**) South Asia, (**e**) Southeast Asia, East Asia, and Oceania, (**f**) North Africa and Middle East, and (**g**) Sub-Saharan Africa. Bars represent total annual DALYs (left y-axis), and the orange line represents the age-standardized DALY rate per 100,000 population (right y-axis). The orange shaded band and gray error bars represent 95% uncertainty intervals (UIs) for projected ASIRs and incident cases, respectively. Pink bars and solid orange lines indicate observed estimates from 1990–2023, whereas teal bars and dashed orange lines indicate BAPC projections for 2024–2040. The vertical dashed line denotes the transition from observed to projected estimates.
