# Supplementary material for: Global Burden and Future Projections of Alopecia Areata: A Global Burden of Disease Study 2023 Analysis
Source: J Clin Med. 2026 Sep 19;15(18):7286. doi: 10.3390/jcm15187286 (PMC13608508; doi:10.3390/jcm15187286)
Supplement: Supplementary file 1 [file jcm-15-07286-s001.zip › Table S1.pdf]

Table S1. Global Burden of Disease (GBD) regional classification of countries.

Region	Country
Central Asia	Armenia
	Azerbaijan
	Georgia
	Kazakhstan
	Kyrgyzstan
	Mongolia
	Tajikistan
	Turkmenistan
	Uzbekistan
Australasia	Australia
	New Zealand
High-income Asia Pacific	Brunei Darussalam
	Japan
	Republic of Korea
	Singapore
Eastern Europe	Belarus
	Estonia
	Latvia
	Lithuania
	Republic of Moldova
	Russian Federation
	Ukraine
Central Europe	Albania

Region	Country
Central Europe (continued)	Bosnia and Herzegovina
	Bulgaria
	Croatia
	Czechia
	Hungary
	Montenegro
	North Macedonia
	Poland
	Romania
	Serbia
	Slovakia
	Slovenia
High-income North America	United States of America
	Canada
	Greenland
Southern Latin America	Argentina
	Chile
	Uruguay
Western Europe	Andorra
	Austria
	Belgium
	Cyprus
	Denmark
	Finland

Region	Country
Western Europe (continued)	France
	Germany
	Greece
	Iceland
	Ireland
	Israel
	Italy
	Luxembourg
	Malta
	Monaco
	Netherlands
	Norway
	Portugal
	San Marino
	Spain
	Sweden
	Switzerland
	United Kingdom
Andean Latin America	Bolivia (Plurinational State of)
	Ecuador
	Peru
Caribbean	Antigua and Barbuda
	Bahamas
	Barbados

Region	Country
Caribbean (continued)	Belize
	Bermuda
	Cuba
	Dominica
	Dominican Republic
	Grenada
	Guyana
	Haiti
	Jamaica
	Puerto Rico
	Saint Kitts and Nevis
	Saint Lucia
	Saint Vincent and the Grenadines
	Suriname
	Trinidad and Tobago
	United States Virgin Islands
Central Latin America	Colombia
	Costa Rica
	El Salvador
	Guatemala
	Honduras
	Mexico
	Nicaragua
	Panama

Region	Country
Central Latin America (continued)	Venezuela (Bolivarian Republic of)
Tropical Latin America	Brazil
	Paraguay
North Africa and Middle East	Afghanistan
	Algeria
	Bahrain
	Egypt
	Iran (Islamic Republic of)
	Iraq
	Jordan
	Kuwait
	Lebanon
	Libya
	Morocco
	Oman
	Palestine
	Qatar
	Saudi Arabia
	Sudan
	Syrian Arab Republic
	Tunisia
	Türkiye
	United Arab Emirates
	Yemen

**Region**	**Country**
South Asia	Bangladesh
	Bhutan
	India
	Nepal
	Pakistan
Southeast Asia, East Asia, and Oceania	China
	Democratic People's Republic of Korea
	Taiwan
	American Samoa
	Cook Islands
	Fiji
	Guam
	Kiribati
	Marshall Islands
	Micronesia (Federated States of)
	Nauru
	Niue
	Northern Mariana Islands
	Palau
	Papua New Guinea
	Samoa
	Solomon Islands
	Tokelau
	Tonga

**Region**	**Country**
Southeast Asia, East Asia, and Oceania (continued)	Tuvalu
	Vanuatu
	Cambodia
	Indonesia
	Lao People's Democratic Republic
	Malaysia
	Maldives
	Mauritius
	Myanmar
	Philippines
	Seychelles
	Sri Lanka
	Thailand
	Timor-Leste
	Viet Nam
Sub-Saharan Africa	Angola
	Central African Republic
	Congo
	Democratic Republic of the Congo
	Equatorial Guinea
	Gabon
	Burundi
	Comoros
	Djibouti

Region	Country
Sub-Saharan Africa (continued)	Eritrea
	Ethiopia
	Kenya
	Madagascar
	Malawi
	Mozambique
	Rwanda
	Somalia
	South Sudan
	Uganda
	United Republic of Tanzania
	Zambia
	Botswana
	Eswatini
	Lesotho
	Namibia
	South Africa
	Zimbabwe
	Benin
	Burkina Faso
	Cabo Verde
	Cameroon
	Chad
	Côte d'Ivoire

**Region**	**Country**
Sub-Saharan Africa (continued)	Gambia
	Ghana
	Guinea
	Guinea-Bissau
	Liberia
	Mali
	Mauritania
	Niger
	Nigeria
	Sao Tome and Principe
	Senegal
	Sierra Leone
	Togo
