# Supplementary material for: Global Burden and Future Projections of Alopecia Areata: A Global Burden of Disease Study 2023 Analysis
Source: J Clin Med. 2026 Sep 19;15(18):7286. doi: 10.3390/jcm15187286 (PMC13608508; doi:10.3390/jcm15187286)
Supplement: Supplementary file 1 [file jcm-15-07286-s001.zip › Table S2.pdf]

Supplementary Table S2. Retrospective validation of Bayesian age-period-cohort model predictive performance.

Region	MAE	RMSE	MAPE (%)	MPE (%)	95% UI Coverage
Global	11837.69	12607.30	0.191	0.191	100
High-income North America	2644.06	2715.13	0.466	-0.466	100
Western Europe	491.02	542.85	0.103	-0.103	100
South Asia	1438.48	1623.08	0.108	-0.108	100
Southeast Asia, East Asia, and Oceania	19488.57	19782.70	1.175	1.175	100
North Africa and Middle East	914.56	1357.29	0.243	0.243	100
Sub-Saharan Africa	268.86	276.39	0.045	-0.045	100

Predictive performance was evaluated by comparing predicted incident cases with observed GBD estimates during the five-year holdout period (2019–2023). Mean absolute error (MAE), root mean square error (RMSE), mean absolute percentage error (MAPE), mean percentage error (MPE), and coverage of the 95% uncertainty intervals (UI) are reported. Positive and negative MPE values indicate overprediction and underprediction, respectively.
