# Supplementary material for: Global Burden and Future Projections of Alopecia Areata: A Global Burden of Disease Study 2023 Analysis
Source: J Clin Med. 2026 Sep 19;15(18):7286. doi: 10.3390/jcm15187286 (PMC13608508; doi:10.3390/jcm15187286)
Supplement: Supplementary file 1 [file jcm-15-07286-s001.zip › Table S3.pdf]

Supplementary Table S3. Incidence patterns by geographical Global Burden of Disease (GBD) Region.

Region	1990 Incident Cases	2023 Incident Cases	2040 Incident Cases (95% UI)	1990 – 2023 Change in incident cases (%)	1990 – 2023 Contribution to Global Change (%)	2023 – 2040 Contribution to Global Change (%)	2023 Age-standardized Incidence Rates
Global	4238528.55	6274991.44	7192851.75 (5409343.54 – 9374774.38)	48.05	100.00	100.00	75.47
Andean Latin America	21651.22	41655.77	50561.40 (35611.86 – 67671.30)	92.39	0.98	0.97	61.45
Australasia	24042.84	38300.79	48518.86 (34840.49 – 65807.27)	59.30	0.70	1.11	111.72
Caribbean	21236.30	30717.76	33933.59 (24652.84 – 46207.70)	44.65	0.47	0.35	61.45

Region	1990 Incident Cases	2023 Incident Cases	2040 Incident Cases (95% UI)	1990 – 2023 Change in incident cases (%)	1990 – 2023 Contribution to Global Change (%)	2023 – 2040 Contribution to Global Change (%)	2023 Age-standardized Incidence Rates
Central Asia	67547.91	98136.23	118803.67 (87721.85 – 159521.25)	45.28	1.50	2.25	98.15
Central Europe	127041.16	118050.77	100318.15 (75010.25 – 130832.50)	-7.08	-0.44	-1.93	97.83
Central Latin America	93648.10	166524.53	183674.99 (137458.03 – 236726.64)	77.82	3.58	1.87	61.58
Eastern Europe	236135.17	218361.05	224553.29 (166845.65 – 295257.74)	-7.53	-0.87	0.67	99.57

Region	1990 Incident Cases	2023 Incident Cases	2040 Incident Cases (95% UI)	1990 – 2023 Change in incident cases (%)	1990 – 2023 Contribution to Global Change (%)	2023 – 2040 Contribution to Global Change (%)	2023 Age-standardized Incidence Rates
High-income Asia Pacific	207329.10	219196.88	196036.22 (152727.46 – 250445.53)	5.72	0.58	-2.52	111.44
High-income North America	511742.66	573812.21	724175.84 (155630.07 – 2083374.27)	12.13	3.05	16.38	141.46
North Africa and Middle East	181174.30	380118.27	461377.88 (352272.39 – 594292.29)	109.81	9.77	8.85	58.59
South Asia	716261.71	1380956.29	1702032.89 (1315204.27 – 2164904.36)	92.80	32.64	34.98	71.04

Region	1990 Incident Cases	2023 Incident Cases	2040 Incident Cases (95% UI)	1990 – 2023 Change in incident cases (%)	1990 – 2023 Contribution to Global Change (%)	2023 – 2040 Contribution to Global Change (%)	2023 Age-standardized Incidence Rates
Southeast Asia, East Asia, and Oceania	1210568.00	1669499.73	1802945.77 (1358646.13 – 2359239.58)	37.91	22.54	14.54	71.01
Southern Latin America	55363.90	84497.44	96935.74 (72267.13 – 127223.66)	52.62	1.43	1.35	112.44
Sub-Saharan Africa	243026.96	626032.47	977642.96 (754457.64 – 1238911.71)	157.60	18.81	38.31	57.06
Tropical Latin America	95059.98	148477.47	154844.48 (118220.66 – 200769.01)	56.19	2.62	0.69	63.91

Region	1990 Incident Cases	2023 Incident Cases	2040 Incident Cases (95% UI)	1990 – 2023 Change in incident cases (%)	1990 – 2023 Contribution to Global Change (%)	2023 – 2040 Contribution to Global Change (%)	2023 Age-standardized Incidence Rates
Western Europe	426511.94	480989.57	484885.85 (377993.15 – 610100.48)	12.77	2.68	0.42	102.08
