# Supplementary material for: Global Burden and Future Projections of Alopecia Areata: A Global Burden of Disease Study 2023 Analysis
Source: J Clin Med. 2026 Sep 19;15(18):7286. doi: 10.3390/jcm15187286 (PMC13608508; doi:10.3390/jcm15187286)
Supplement: Supplementary file 1 [file jcm-15-07286-s001.zip › Table S4.pdf]

Table S4. Decomposition results of change in incident cases, 1990-2023.

Region	Population Growth	Population Aging	Epidemiologic Change	Net Change
High-income North America	184886.97	1158.31	-123924.69	62120.59
Western Europe	64662.01	-6393.55	-3834.58	54433.88
South Asia	498593.53	139658.12	26380.32	664631.97
Southeast Asia, East Asia, and Oceania	394169.20	138218.40	-73455.80	458931.80
North Africa and Middle East	179467.64	40719.18	-21286.65	198900.17
Sub-Saharan Africa	346385.65	28705.79	7931.87	383023.31
