# Supplementary material for: Global Burden and Future Projections of Alopecia Areata: A Global Burden of Disease Study 2023 Analysis
Source: J Clin Med. 2026 Sep 19;15(18):7286. doi: 10.3390/jcm15187286 (PMC13608508; doi:10.3390/jcm15187286)
Supplement: Supplementary file 1 [file jcm-15-07286-s001.zip › Table S5.pdf]

Table S5. Pairwise Pearson correlation coefficients describing similarity of age-specific incidence profiles between six Global Burden of Disease (GBD) regions in 1990 and in 2023.

Year	Region	Southeast Asia, East Asia, and Oceania	High-income North America	North Africa and Middle East	Western Europe	Sub-Saharan Africa	South Asia
**1990**	**Southeast Asia, East Asia, and Oceania**	1.000	0.985	1.000	0.997	1.000	1.000
	**High-income North America**	0.985	1.000	0.983	0.991	0.984	0.985
	**North Africa and Middle East**	1.000	0.983	1.000	0.996	1.000	1.000
	**Western Europe**	0.997	0.991	0.996	1.000	0.996	0.997
	**Sub-Saharan Africa**	1.000	0.984	1.000	0.996	1.000	1.000
	**South Asia**	1.000	0.985	1.000	0.997	1.000	1.000
**Year**	**Region**	**Southeast Asia, East Asia, and Oceania**	**High-income North America**	**North Africa and Middle East**	**Western Europe**	**Sub-Saharan Africa**	**South Asia**
**2023**	**Western Europe**	1.000	0.988	0.994	0.994	0.993	0.993
	**High-income North America**	0.988	1.000	0.992	0.992	0.991	0.991
	**Southeast Asia, East Asia, and Oceania**	0.994	0.992	1.000	1.000	1.000	1.000
	**South Asia**	0.994	0.992	1.000	1.000	1.000	1.000
	**North Africa and Middle East**	0.993	0.991	1.000	1.000	1.000	1.000
	**Sub-Saharan Africa**	0.993	0.991	1.000	1.000	1.000	1.000
